# Editorial: The use of growth promoters and their alternatives in livestock production

**DOI:** 10.3389/fvets.2022.945308

**Published:** 2022-07-25

**Authors:** Manuel Gonzalez Ronquillo, Einar Vargas-Bello-Pérez

**Affiliations:** ^1^Departamento de Nutricion Animal, Facultad de Medicina Veterinaria y Zootecnia, Instituto Literario 100 Ote, Universidad Autonoma del Estado de Mexico, Toluca, Mexico; ^2^Animal Production Group, Faculty of Environmental and Agricultural Sciences, University of Salamanca, Salamanca, Spain; ^3^Department of Animal Sciences, School of Agriculture, Policy and Development, University of Reading, Reading, United Kingdom

**Keywords:** ruminant, poultry, pigs, secondary metabolites, antimicrobial resistance, enzymes

This Research Topic aimed at compiling papers suitable to improve our knowledge and understanding of “The Use of Growth Promoters and their Alternatives in Livestock Production”. The effects of the use of growth promoters began in the 1940s when chickens were fed feed containing tetracycline fermentation by-products. In this case, chickens showed higher growth rates than chickens that were not fed feed containing antibiotics (1). Since then, the use of synthetic growth promoters (SGP; e.g., antibiotics, beta-agonists) or natural growth promoters (NGP: e.g., plant extracts, essential oils, enzymes derived from fungi, exogenous enzymes, direct feed microbials, prebiotics, phytobiotics, guanidinoacetic acid, spirulina, algae-derived polysaccharides, and synbiotic) (Figure 1) has expanded in their use in various animal species.

**Figure 1:**
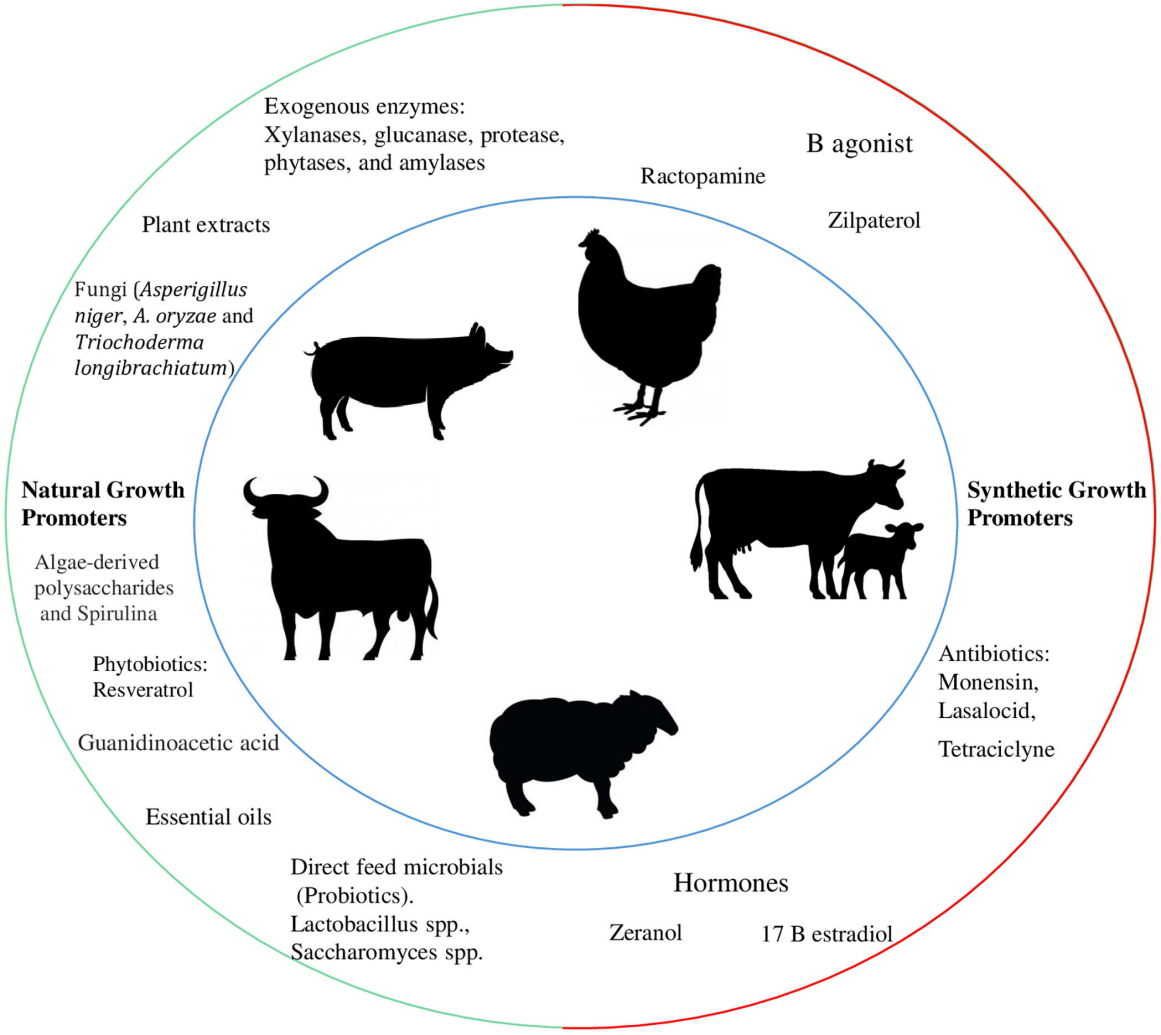
Most common natural and synthetic growth promoters used in livestock production. Red line = banned in some countries, due to antimicrobial resistance, or harm to human and animal health (2).

Although natural and synthetic growth promoters have been widely used in animal production, in recent decades to improve their production parameters, there is strong evidence linking the presence of residues of synthetic growth promoters (i.e., antibiotics, anabolic compounds, hormones, and beta-agonists) in feed components and animal diets to negative effects in both human and animal health (3, 4).

In this special electronic collection, there are eight articles covering the aforementioned aspects. Rafiq et al. reported the role of different growth enhancers as alternatives to in-feed antibiotics in the poultry industry, and this review article highlighted the advantages of using biological products instead of antibiotics as poultry in-feed growth enhancers for improving production performance, reducing intestinal pathogenic bacteria, maintaining gut health, potentiate immune response, and the safety and wholesomeness of meat and eggs, as well as showing improved safety of poultry products for human consumption.

Feed additives have gained popularity in poultry production for having many advantages without leaving any residues in poultry products. Nusairat and Wang fed broilers with xylanases as a replacement for antibiotics (i.e., Bacitracin) and reported an improved feed conversion ratio (FCR, *P* = 0.0001) from 1 to 42 d compared to the negative control diet (NC, low-energy broiler diets). The NC+xylanases reduced energy utilization in broilers raised to market weight, compared to a standard diet and NC, concluding that a blend of xylanase (10 XU/g feed) and *Bacillus spp*. (1 × 105 CFU/g feed) can be used as an alternative to antibiotic growth promoters (i.e., Bacitracin) in low-energy broiler diets. Liu et al. studied the inclusion of algae-derived polysaccharides (ADP) as a growth promoter in broiler chickens, concluding that dietary ADP exerted beneficial effects on growth performance, antioxidant capacity, and gut health in broilers. Similarly, Chang et al. investigated the effects of mesobiliverdin IX alfa (MBV)-enriched microalgae spirulina extracts on growth performance, blood parameters, intestinal morphology, and gut microbiota of broilers, and revealed that MBV-enriched microalgae spirulina extracts improved intestinal health and benefit microflora composition of broilers, without affecting animal performance (*P* > 0.05) (i.e., live weight, average daily gain and feed efficiency). These results demonstrate that the use of NGP can replace the use of antibiotics, thus decreasing the effects of AMR.

In pigs, the effect of including tea tree oil (TTO) added as TTO capsulation or un-encapsulated on growth performance, antioxidant capacity, and intestinal microbiome of weaned pigs has been reported by Wang et al. They showed that TTO improves the growth performance of weaned pigs and further showed that encapsulation of TTO was superior to its unencapsulated counterpart. Encapsulated TTO was similar to the positive control (i.e., antibiotics supplemented) group and could be a potential alternative to the use of antibiotics in weaned pigs, without the risk of AMR. Another study using pigs (Chen et al.) investigated the effect of dietary *Yucca schidigera* extract (YSE) supplementation on animal performance, nutrient digestibility, and ammonia emission in manure from sows. They reported that supplementation of YSE in sow diets during gestation and lactation stages resulted in trends toward a reduction in the number of stillbirth piglets (*P* = 0.08), weak piglets (*P* = 0.06), pre-weanling mortality, and diarrhea events, partly explained by improved nutrient digestibility, and reduce nitrogen losses from sows.

In ruminants, the inclusion of growth promoters has been widely studied, in the present special issue. For example, Li et al. determined the effects of supplementing Jinjiang bulls with guanidinoacetic acid (GAA) and concluded that adding 0.2% GAA into the diet improves the average daily gain and decreases the value of feed to gain ratio resulting in an improved meat quality by increasing a^*^(redness), and b^*^ (yellowness) values, and contents of creatine kinase, muscle glycogen, creatinine, and laminin in *Longissimus dorsi*. Likewise, Song et al. determined the effects of yeast (*Saccharomyces cerevisiae*) culture (YC), added to pelleted total mixed rations (TMR) at two proportions of corn in the diet of fattening lambs. Their results showed that live yeast cells could not survive during pelleting, and thus any biological effects of the YC were the result of feeding dead yeast and the metabolites of yeast fermentation rather than live yeast cells, however, this study indicated that YC products can be supplemented to pelleted TMR for improving lamb growth performance, as a consequence of improved fiber digestibility, which coincides with Kerr and Shurson (5), who reported that an enzyme may not only need to match a target substrate(s), but a “cocktail” of enzymes may also be necessary to efficiently break down complex fibrous carbohydrate matrices in order to alleviate the negative impact of these compounds on nutrient digestibility or voluntary feed intake and thus improve energy digestibility or voluntary feed intake, thus being metabolically and economically beneficial for pig production.

The indiscriminate use of antibiotics might result in the deposition of residues in animal food products and develop antimicrobial resistance (2), therefore, both in humans and animals, many diseases are becoming difficult to treat, and many studies have shown that antibiotics administered to livestock are poorly absorbed through the gut and usually excreted without being metabolized. These excreted antibiotics eventually accumulate in the environment (6) and enter the human food chain resulting in bioaccumulation of drug residues in the human body. As a result, the inclusion of natural growth promoters (i.e., direct feed microbials, prebiotics, phytobiotics, spirulina, synbiotics, and their combination) has been used more frequently in livestock production, with the advantage of not producing antimicrobial resistance, with no environmental risk from its excretion into the environment.

Considering the current scenario related to AMR, this Research Topic contributes to the knowledge on the addition of growth promoters and nutritional strategies to make animal production safer, more efficient, and sustainable, with less environmental impact.

## Author contributions

MG wrote the first draft. EV-B-P wrote and edited the second draft. All authors contributed to the article and approved the submitted version.

## Funding

MG enjoyed his sabbatical leave at the University of Salamanca, Spain funded by Universidad Autonoma del Estado de Mexico. EV-B-P was supported by a visiting scholarship of the Universidad Autonoma del Estado de México (Project ID UAEMex4974/2020).

## Conflict of interest

The authors declare that the research was conducted in the absence of any commercial or financial relationships that could be construed as a potential conflict of interest.

## Publisher's note

All claims expressed in this article are solely those of the authors and do not necessarily represent those of their affiliated organizations, or those of the publisher, the editors and the reviewers. Any product that may be evaluated in this article, or claim that may be made by its manufacturer, is not guaranteed or endorsed by the publisher.
